# Perioperative enfortumab vedotin plus pembrolizumab in muscle-invasive bladder cancer: unprecedented outcomes from KEYNOTE-B15

**DOI:** 10.1093/oncolo/oyag332

**Published:** 2026-08-19

**Authors:** Sarah Azari, Amirali Salmasi, Tyler Stewart

**Affiliations:** Department of Urology, University of California San Diego, La Jolla, CA 92093, United States; Department of Urology, University of California San Diego, La Jolla, CA 92093, United States; Department of Medicine, University of California San Diego, La Jolla, CA 92093, United States

**Keywords:** bladder cancer, enfortumab vedotin, pembrolizumab, perioperative therapy, bladder preservation

For the last 20 years, cisplatin-based chemotherapy has been standard-of-care for patients with muscle invasive bladder cancer (MIBC) before cystectomy. Despite survival benefit, neoadjuvant chemotherapy has been infrequently used due to eligibility and preference.^1^^,^^2^ The recent KEYNOTE(KN)-905 and KN-B15 studies have shifted this paradigm by introducing enfortumab vedotin plus pembrolizumab (EV-P) in the perioperative space. EV-P first demonstrated efficacy in locally advanced or metastatic urothelial cancer in EV-302, where it improved progression-free and overall survival (OS) compared to platinum-based chemotherapy, with a 30% complete response rate.^3^ KN-905/EV-303 then evaluated perioperative EV-P with cystectomy versus cystectomy alone for patients with MIBC who are ineligible or refuse cisplatin. KN-905 demonstrated substantial improvement in event-free survival (EFS) and OS with a pathologic complete rate (pCR) of 57%.^4^ These studies set the stage for KN-B15.

KN-B15 was a large (*N* = 808) global phase 3 trial of patients with MIBC (T2-T4aN0/N1)M0 randomized to neoadjuvant gemcitabine plus cisplatin versus perioperative EV-P (4 cycles of neoadjuvant EV-P followed by 5 cycles of adjuvant EV and 13 cycles of adjuvant P). The primary endpoint was EFS with secondary endpoints including OS, pCR, and safety. In this study, 87% and 90% of patients in the EV-P and cisplatin arms received surgery, respectively. Adjuvant therapy was started in 65% of patients in the EV-P arm, and 51% completed the scheduled regimen.

EV-P significantly improved EFS and OS compared to cisplatin-based chemotherapy (EFS HR: 0.53 (0.41-0.70); 2 yr EFS rate 79% vs 66%; OS HR: 0.65 (0.48-0.89); 2 yr OS: 87% vs 81%), with a pCR rate of 56% (64% of those that underwent cystectomy). Grade ≥3 treatment-emergent adverse events were more frequent with EV-P (75.7% vs 67.2%).

The trial does have limitations. First, data emerged during the trial making adjuvant and perioperative immunotherapy new standards. Although adjuvant immunotherapy was permitted in the cisplatin arm if clinically indicated, only 7% received it. Second, the amount of time on treatment was significantly longer in the EV-P arm (12 weeks of cisplatin-gemcitabine vs 27 weeks of EV-P and 24 additional weeks of P), doubling the time on cytotoxic chemotherapy. This increases treatment toxicity as well as financial and time burdens. Clinicians are left wondering if similar results could be obtained with fewer cycles.

Despite these limitations, KN-905 and KN-B15 provide a strong rationale for changing the standard of care in MIBC. For the first time, a non-cisplatin regimen has outperformed cisplatin-based neoadjuvant chemotherapy, positioning EV-P as a new standard for patients with MIBC regardless of cisplatin-eligibility.

These findings also raise important questions regarding implementation. First, the optimal duration and necessity of adjuvant therapy remain unclear. Currently, completion of the full perioperative regimen is reasonable to maximize the chance of cure, although future studies may support de-escalation. Biomarkers such as ctDNA may help in tailoring intensity of adjuvant therapy. Support for a ctDNA guided approach in urothelial cancer management comes from IMvigor011, which demonstrated improved disease-free survival with adjuvant atezolizumab compared to placebo among ctDNA-positive patients following cystectomy. Outcomes remained favorable among ctDNA-negative patients without adjuvant therapy. These findings highlight the potential utility of ctDNA for risk stratification and treatment selection in urothelial cancer.^5^ Another trial which may help elucidate the role of adjuvant EV is the VOLGA trial, which is investigating perioperative EV plus durvalumab with or without tremelimumab for patients with MIBC who are cisplatin-ineligible compared to cystectomy alone. After cystectomy Arms 1 and 2 will receive adjuvant immunotherapy, and Arm 3 will undergo observation; no EV is given post-cystectomy.^6^ This may provide insight into the contribution of adjuvant EV. Further prospective studies are currently in design to explore de-escalation strategies.

A critical finding with possible implications for treatment strategies is the pCR rate which has renewed interest in bladder preservation. With pCR rates of 57% and 56% respectively in KN-905 and KN-B15, there is growing interest in strategies to defer surgery in carefully selected patients. Studies to investigate the possibility of alternatives, such as chemoradiation, intravesical therapy, or even surveillance are ongoing (Table 1). The role of imaging, biopsy, genomics, and biomarkers in selecting patients who may be able to avoid cystectomy is another area of investigation. In the meantime, clinicians are may face increased demand from patients for bladder preservation without any current data to guide management.

**Table 1.** Selected ongoing trials evaluating EV-P based bladder preservation strategies.

Trial	Name	Design	Primary endpoint
**NCT06470282 (EV-PRIME)**	Enfortumab Vedotin and Pembrolizumab Combined With Radiotherapy in Muscle Invasive Bladder Cancer	Phase Ib dose-escalation study of EV-P with concurrent radiation, followed by a Phase II expansion of EV-P + radiation	Safety (phase Ib), clinical CR (phase II)
**NCT07475806**	A Study to Find Out if Enfortumab Vedotin Given With Pembrolizumab Helps People With Muscle-invasive Bladder Cancer Keep Their Bladder	Single arm trial of EV-P as a bladder preserving strategy	Clinical CR, bladder intact EFS
**NCT06809140 (HCRN GU22-598)**	Enfortumab Vedotin Plus Pembrolizumab With Selective Bladder Sparing for Treatment of Muscle-invasive Bladder Cancer	Single arm trial of induction EV-P followed by restaging and maintenance EV-P for patients with clinical CR	Clinical CR

Although EV-P will become the new standard for most, further data is likely to emerge about subsets of patients with MIBC that may not benefit from EV-P. Further work is needed to ensure that patients whose cancer contains histologic variants such as plasmacytoid and micropapillary benefit from EV-P. A retrospective analysis of the real-world UNITE study demonstrated efficacy of EV across multiple histologic variants however efficacy appeared to decrease with increasing proportion of variant histology component. Limited activity was observed in tumors with neuroendocrine histology and in cases of pure variant histology.^7^ Prospective studies are needed to further define the role of EV in these patient populations.

Lastly, the incorporation of EV-P in the perioperative setting also raises the question of sequencing and how to manage patients who progress after receiving perioperative EV-P. It is unclear if additional antibody-drug conjugates will prove beneficial for these patients and what the optimal salvage regimen will be. Early results from the phase I NEXUS-01 study demonstrated promising activity of LY4052031, a Nectin-4–targeted antibody-drug conjugate which utilizes a distinct payload from EV, in advanced urothelial carcinoma. Importantly, responses were observed in patients previously treated with EV, suggesting that Nectin-4 may remain a therapeutically relevant target after EV exposure. The observed activity in EV treated patients is consistent with preclinical data suggesting that resistance may be mediated by the payload.^8^

EV-P is the first antibody-drug conjugate to demonstrate improved perioperative outcomes, but it is unlikely to be the last. KN-B15 marks a clear inflection point and establishes a new foundation for perioperative therapy in bladder cancer. As EV-P moves toward standard-of-care, future work will be critical in refining patient selection, defining the necessary components and duration of therapy, and identifying opportunities for de-escalation.
